# 

**Published:** 1889-04

**Authors:** 


					﻿io cts. a copy
APRIL, 1889.
$ i .00 per year.
IJAl.I.s
eJovKNAL'J
HE Al T H
ESTABLISHED 18^4
IK 36
c/V 2. 4.
OFFICE: 206 BROADWAY, EVENING POST BUILDING, Room 11. N. Y.
Entered as Second-class Matter at the Post-Office, New York.
				

